# CAD–CAM Complete Dentures Manufactured Using Additive and Subtractive Manufacturing Techniques: A Feasible Clinical Approach for Managing Geriatric Patients With Advanced Residual Ridge Resorption

**DOI:** 10.1155/crid/9813868

**Published:** 2025-09-26

**Authors:** Silvia Rojas-Rueda, Franciele Floriani, Salahaldeen Abuhammoud, Ali Mohammed, Kelvin I. Afrashtehfar, Carlos A. Jurado

**Affiliations:** ^1^Division of Dental Biomaterials, Department of Clinical and Community Sciences, School of Dentistry, The University of Alabama at Birmingham, Birmingham, USA; ^2^Department of Prosthodontics, The University of Iowa College of Dentistry and Dental Clinics, Iowa City, Iowa, USA; ^3^Evidence-Based Practice Unit, Department of Clinical Sciences, Ajman University College of Dentistry, Ajman City, UAE; ^4^Department of Reconstructive Dentistry and Gerodontology, School of Dental Medicine (ZMK), Faculty of Medicine, University of Bern, Berne, Switzerland; ^5^Oral Implantology Research Institute (OIRI), Dubai, UAE; ^6^Specialist Prosthodontist, West Vancouver, Canada; ^7^Division of Operative Dentistry, Department of General Dentistry, The University of Tennessee Health Science Center College of Dentistry, Memphis, Tennessee, USA; ^8^Ponce Health Sciences University School of Dental Medicine, Ponce, Puerto Rico

## Abstract

Edentulous geriatric patients represent a challenging clinical scenario for complete denture therapy. Factors including atrophic alveolar ridges, cognitive impairments, and anxiety are common and can complicate complete denture therapy. Computer-aided design and computer-aided manufacturing (CAD–CAM) technologies offer different workflows for fabricating complete dentures. This clinical case report series describes two digital workflows used for the fabrication of digital complete dentures. These procedures reduced the number of appointments and expedited manufacturing and delivery. The techniques presented require three to four appointments, fewer appointments compared to the traditional protocol used for complete dentures.

## 1. Introduction

Computer-aided design and computer-aided manufacturing (CAD–CAM) technologies have transformed several health-related fields by improving the precision and predictability of treatments. In prosthodontics, CAD–CAM technology has modernized the design and fabrication of complete dentures, offering significant improvements over traditional techniques [1]. CAD–CAM technologies permit visualizing and modifying the prosthetic design digitally within the computer program [2]. This facilitates fine-tuning the prosthesis to ensure it satisfies the patient's and clinicians' expectations prior to its transfer to the computer-aided manufacturing (CAM) system of preference for its production.

Two main methods are commonly used for manufacturing dental prostheses using digital technologies: additive manufacturing (a-CAM), which includes technologies like 3D printing and sintering, and subtractive manufacturing (s-CAM), primarily associated with milling processes [3–5]. The evolution of these technologies traces back to historical milestones, such as the development of precision milling by the industrialist John Wilkinson in 1774 and the invention of stereolithography by Charles Hull in 1983 [5, 6]. More recently, these technologies have gained popularity in dentistry. For example, CAD–CAM removable complete dentures provide several advantages over traditional manufacturing techniques, including increased patient satisfaction, reduced costs, less material wastage, improved denture base adaptation, enhanced retention, and the possibility of digital archiving [5, 7]. Additionally, research suggests that these prostheses require fewer postoperative visits, appealing to patients with limited access to oral care [6]. Furthermore, the digital fabrication of complete dentures, with contemporary 3D printing systems, has shown to be up to 65% more cost-effective compared to traditional techniques, owing to lower material costs and reduced time spent in laboratory and chairside [8, 9]. Additionally, less discomfort has been reported after the delivery of 3D-printed dentures compared to conventional dentures, although no significant differences have been observed in maintenance or postinsertion repairs [10, 11].

However, the application of CAD–CAM technologies in prosthodontics is not without challenges. Studies have shown mixed results in the accuracy and consistency of complete dentures produced using additive and subtractive manufacturing techniques, hence emphasizing the importance of understanding how the material properties and manufacturing processes impact the final dental prostheses [8–10]. In a recent study, the accuracy of s-CAM, injection molded, and compression molded bases was more accurate than those produced with a-CAM technologies [9, 10]. In contrast, Hwang found that the trueness of a maxillary denture base produced with digital light processing (DLP) 3D printing technologies was superior to that of a milled prosthesis [11]. Research suggests that the accuracy of 3D-printed dentures may be affected by many variables, including the object geometry, the photopolymer resin used, and the degree of distortion occurring during postcuring. Alharbi et al. explored these variables and confirmed that the accuracy and efficiency of the manufacturing process vary between 3D printers and that software-related light source parameters affect the accuracy and strength of the 3D-printed object [12]. Similarly, Jin et al. noted that the effect of different build angles on the tissue surface of complete denture bases is unknown but suggested that, although there are some differences noted, they are not statistically significant [13]. This contrasts with another study where the printing orientation of test a-CAM material specimens affected the printing accuracy, flexural strength, roughness, and response to common intraoral microorganisms such as *Candida albicans*. Although the wear characteristics and fracture strength of 3D-printed teeth compared to highly crosslinked denture teeth are often mentioned as a disadvantage, studies have demonstrated that 3D-printed artificial teeth are adequate for clinical use [14].

The importance of advancing CAD–CAM technology in dentistry is emphasized by the growing elderly population, the global prevalence of edentulism, and the limitations of traditional complete dentures in addressing the functional and esthetic needs of this group of patients who often suffer from unfavorable intraoral and systemic conditions. This clinical case series report introduces two digital workflows that have been successfully applied to treat elderly patients with severe residual ridge resorption. These methods effectively utilize both additive and subtractive manufacturing techniques to create prostheses with satisfactory retention, stability, and support.

## 2. Case Series Presentation

### 2.1. Patient 1

A 74-year-old male patient presented with the chief complaint of wanting a new set of complete dentures after losing his previous set of complete dentures a year ago. After a clinical evaluation, the patient was diagnosed with functional impairment related to maxillary and mandibular complete edentulism. Intraorally, significantly resorbed maxillary and mandibular residual ridges were observed. He was offered various treatment options, including implant overdentures, complete arch fixed implant-supported prosthesis, and complete dentures. Due to financial constraints and ease of maintenance, the patient selected the complete dentures. He was informed about the alternative of fabricating the prosthesis using a combination of digital and analog techniques to shorten the number of appointments. The patient expressed interest and signed the informed consent and treatment plan forms. Additionally, preliminary impressions with irreversible hydrocolloid (Jeltrate Fast, Dentsply Sirona, Charlotte, North Carolina, United States) were made at the end of the first appointment.

**Figure 1:**
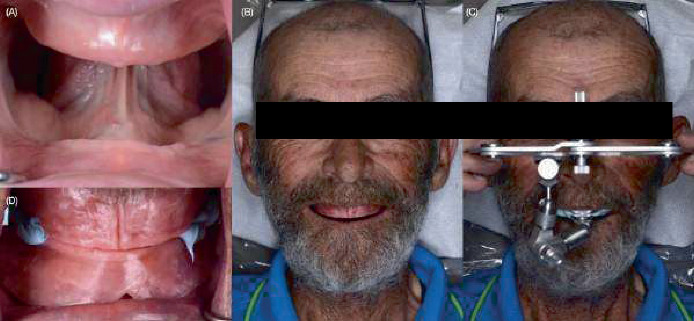
Initial situation and maxillomandibular relationship records. (A) Initial intraoral situation, (B) frontal photograph smiling with occlusion rims, (C) arbitrary face bow record, and (D) jaw relation record with wax rims.

On the second appointment, definitive impressions were made using light-bodied polyvinyl siloxane (PVS) impression material (Aquasil Ultra, Dentsply Sirona, Charlotte, North Carolina, United States) and customized impression trays fabricated from the preliminary impressions made previously. Maxillary and mandibular definitive casts were poured using fast-setting type IV stone (Snap Stone, Whip Mix, Louisville, Kentucky, United States), and occlusion rims (Bite Blocks, Keystone Industries, Gibbstown, New Jersey, United States) were fabricated on the definitive casts by the clinician after the second appointment. Subsequently, the lip support, vertical dimension of occlusion, the smile line, and maxillomandibular relationships were recorded in centric relation, and an arbitrary face bow record (Sam 3, Great Lakes Dental Technologies, Tonawanda, New York, United States) (Figure 1) was used to articulate the definitive casts on a semiadjustable articulator (Sam 3, Great Lakes Dental Technologies, Tonawanda, New York, United States). Additionally, at the end of the appointment, the artificial tooth form and shade were selected based on the patient's preferences.

**Figure 2:**
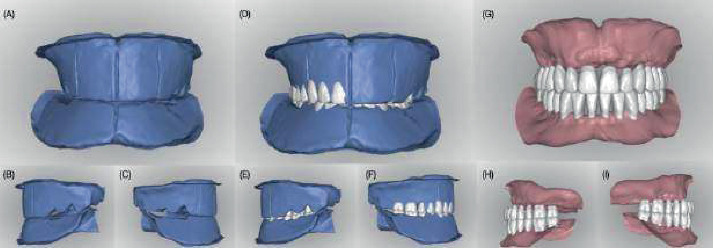
Occlusion rims and digital artificial tooth arrangements. (A–C) Scanned occlusion rims. (D–F) Digital artificial tooth arrangements over the scanned occlusion rims. (G–I) Artificial tooth arrangement and residual ridge relationships obtained from scanning the definitive maxillary and mandibular casts.

**Figure 3:**
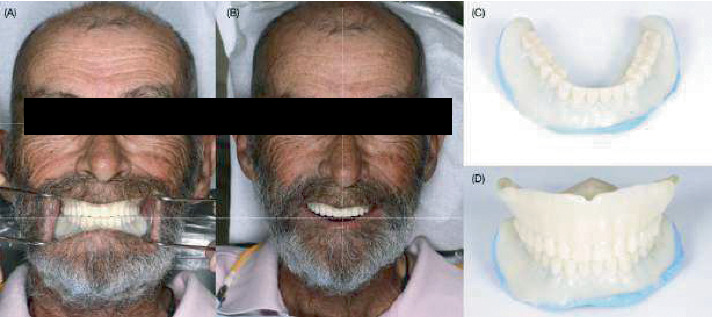
Try-in complete dentures. (A) Extraoral photograph with retracted lips, (B) extraoral photograph of the patient smiling, (C) mandibular try-in complete denture relined with PVS impression material, and (D) maxillary and mandibular relined try-in complete dentures.

**Figure 4:**
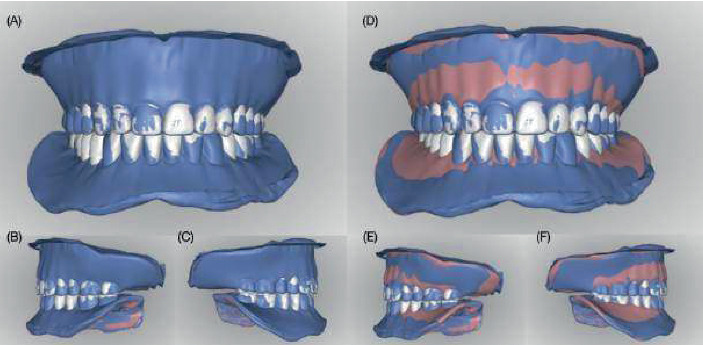
Digital design of the definitive complete dentures. (A–C) New digital artificial tooth arrangement over the previous design. (D–F) Full digital design and tooth arrangement for the new set of complete dentures (pink and white colored) overlying the previous digital design (blue colored).

The occlusion rims and master casts were scanned (D1000, 3Shape, Copenhagen, Denmark), and the file was outsourced for the fabrication of try-in and definitive prostheses (AvaDent, Scottsdale, Arizona, United States) (Figure 2). The company (AvaDent, Scottsdale, Arizona, United States) mailed a set of 3D-printed try-in complete dentures. On the third appointment, the patient and clinician evaluated the esthetics, phonetics, lip support, smile line, retention, and occlusion with the try-in complete dentures. At this stage, the patient expressed being satisfied with the esthetic outcome. However, a wash with light-body PVS material (Aquasil Ultra, Dentsply Sirona, Charlotte, North Carolina, United States) was done as a reline for the lower denture to improve its adaptation (Figure 3). After the fit, esthetics, and function of the try-in complete dentures were deemed adequate, the definitive prostheses were manufactured following the contours of the try-in complete dentures (Figure 4).

**Figure 5:**
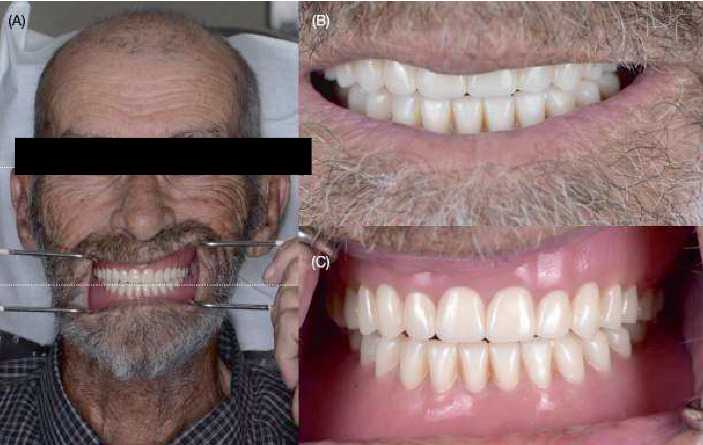
Final complete dentures. (A) Extraoral photographs with retracted lips, (B) smile photograph, and (C) intraoral photograph.

On the fourth appointment, the definitive milled CAD–CAM complete dentures were delivered. Their fit was evaluated on the articulator, accounting for any minor distortions occurring during manufacturing. Subsequently, the prostheses were tried into the patient's mouth, and the retention, midline, esthetics, phonetics, lip support, vertical dimension of occlusion, and centric relation were evaluated and deemed satisfactory by the patient and the clinician (Figure 5). The definitive milled CAD–CAM complete dentures in this study were fabricated entirely from a single prepolymerized polymethylmethacrylate (PMMA) puck (monolithic dentures), in which both the denture base and teeth were milled together as one piece. No separate milling and bonding of the denture base and teeth were performed.

### 2.2. Patient 2

An 82-year-old patient presented with the chief complaint of needing complete dentures. He lived in a remote area with a shortage of dental care. Due to commuting difficulties, he requested the fabrication of complete dentures in the minimum number of appointments possible. After the initial intraoral evaluation, he was diagnosed with complete edentulism with severely atrophic maxillary and mandibular residual ridges. Medical history was reviewed, and no systemic diseases were reported. The patient was classified as ASA Class I, suggesting a healthy systemic status. The patient was given the option of CAD–CAM complete dentures to expedite the process, and he accepted. The patient signed the treatment plan, and preliminary impressions were made using dual-phase irreversible hydrocolloid (AccuDent, Ivoclar Group, Schaan, Liechtenstein). Preliminary impressions were used to fabricate customized impression trays made with PMMA resin.

**Figure 6:**
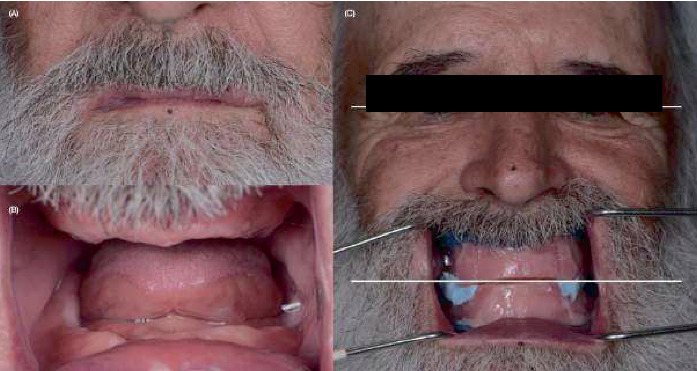
Initial situation. (A) Extraoral photograph, (B) intraoral photograph, and (C) maxillomandibular relationships record with occlusion rims.

**Figure 7:**
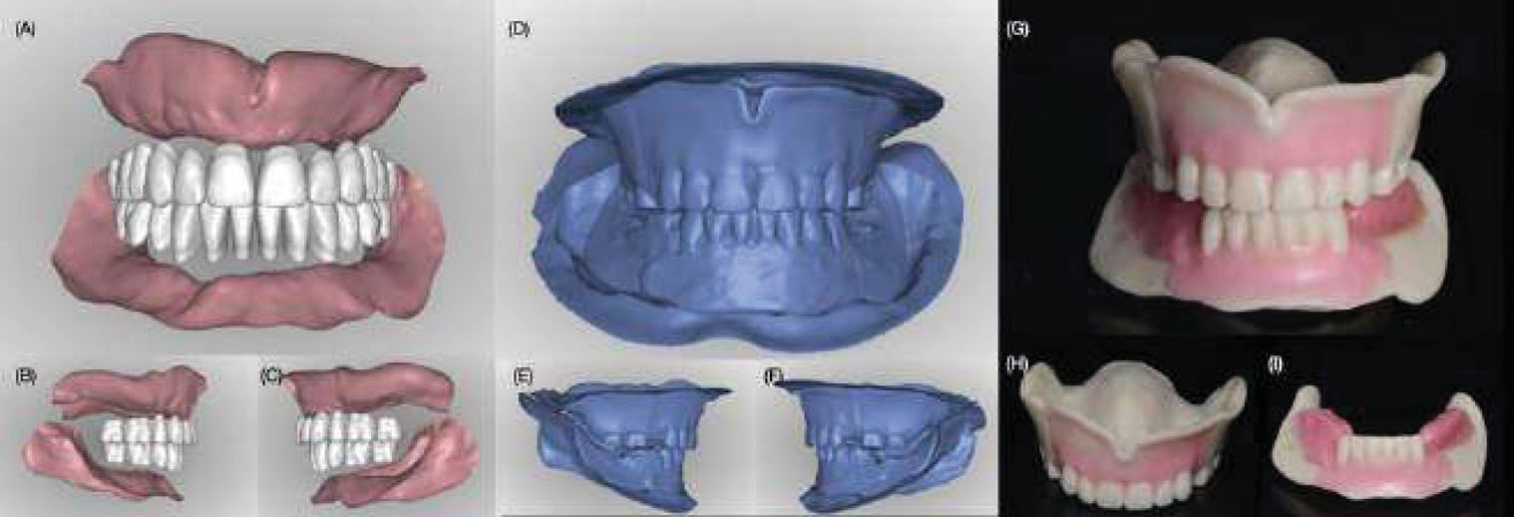
Artificial tooth arrangements and try-in complete dentures. (A–C) Digital artificial tooth arrangements and scanning of residual ridges. (D–F) Digital artificial tooth arrangements. (G–I) 3D-printed artificial tooth arrangements with posterior occlusion rims.

On the second appointment, definitive impressions were made conventionally using PVS impression material (Aquasil Ultra, Dentsply Sirona, Charlotte, North Carolina, United States) with the customized impression trays. Additionally, maxillary and mandibular definitive casts were fabricated with Type IV dental stone (Snap Stone, Whip Mix, Louisville, Kentucky, United States). Maxillomandibular relationship records were made with occlusion rims (Bite Blocks, Keystone Industries, Gibbstown, New Jersey, United States), and the smile line, lip support, and occlusal plane were assessed with the occlusion rims (Figure 6). Tooth mold and shade were selected by the patient and clinician, and a face bow record was made. Subsequently, the definitive casts were mounted in a semiadjustable articulator (Sam 3, Great Lakes Dental Technologies, Tonawanda, New York, United States), and the master casts were scanned. The definitive casts and the occlusion rims were scanned, and their respective digital files were outsourced for the fabrication of 3D-printed try-in complete dentures, with the anterior teeth arrangement set up in wax (AvaDent, Scottsdale, Arizona, United States) (Figure 7).

**Figure 8:**
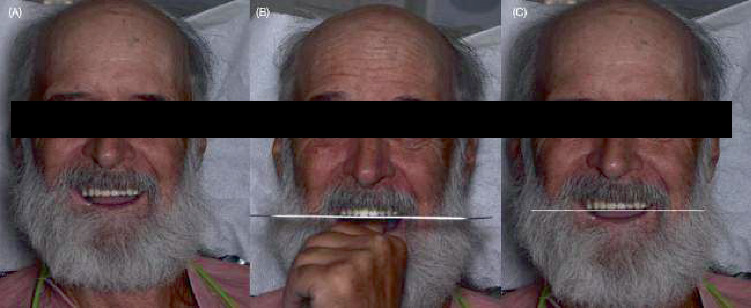
Evaluation of artificial tooth arrangements. (A) Frontal photograph of the patient smiling. (B) Evaluation of occlusal plane with the fox plane. (C) Relationship of occlusal plane with interpupillary line.

**Figure 9:**
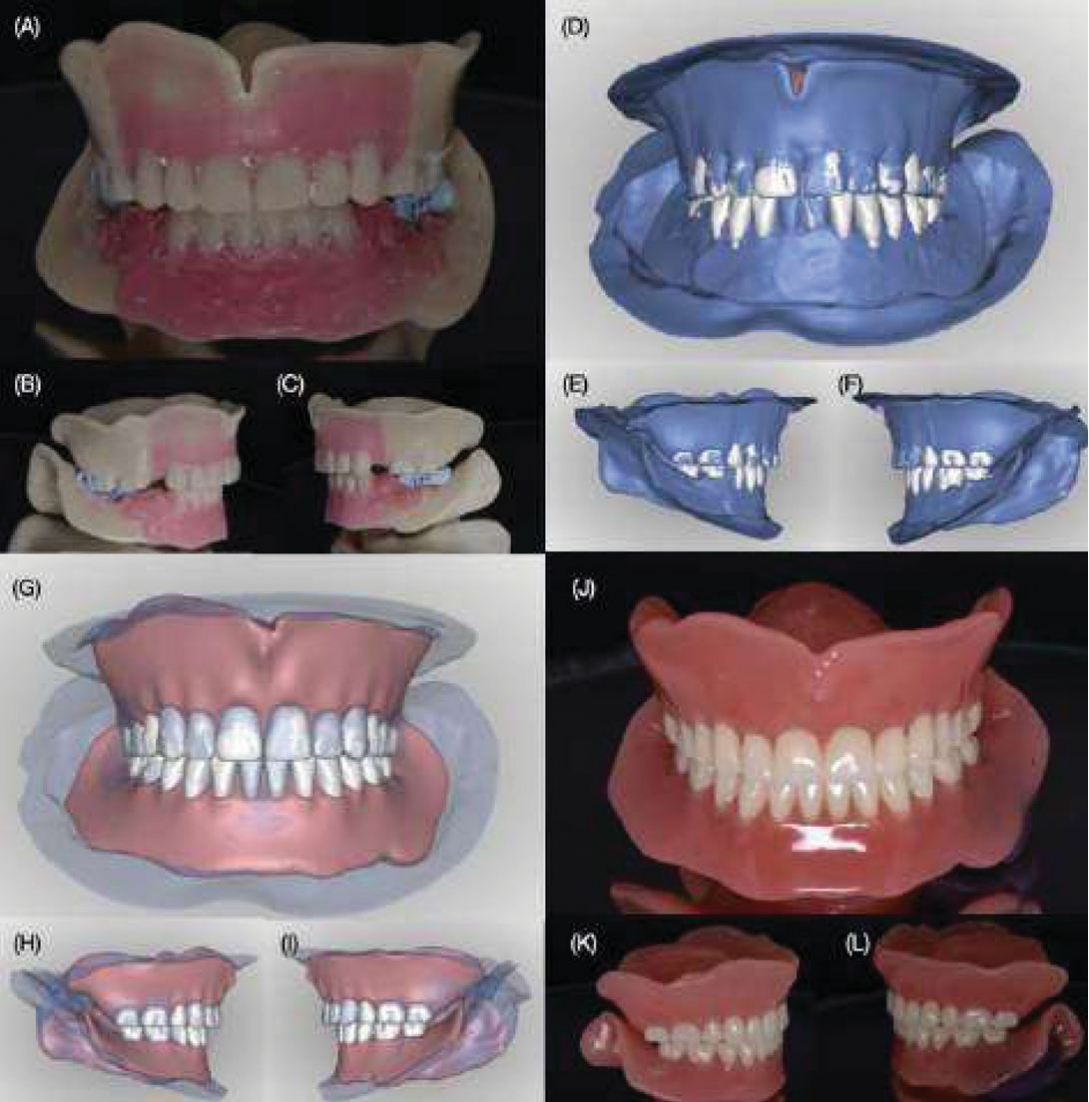
Definitive complete dentures. (A–C) 3D-printed try-in complete dentures related to PVS impression material. (D–F) New digital artificial tooth arrangements overlying the scan from 3D-printed try-in complete dentures. (G–I) Digital designs of the definitive complete dentures. (J–L) 3D-printed CAD–CAM complete dentures.

**Figure 10:**
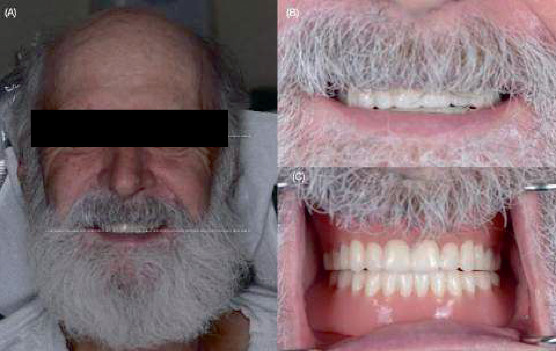
3D-printed complete dentures. (A) Extraoral frontal photograph. (B) Photograph of the patient smiling. (C) Intraoral photograph of 3D-printed complete dentures.

On the third appointment, the lip support, midline, smile line, buccal corridors, phonetics, and occlusal plane were evaluated using Wagner Try-In (WTI) dentures, and esthetic approval was obtained by the patient (Figure 8). In our protocol, the “WTI” refers to a digital try-in design where only the anterior teeth are printed in a tooth-colored resin as part of the same structure with the denture base, allowing for the evaluation of esthetics, midline, and smile line without committing to the final tooth arrangement. In this case, the anterior teeth were printed as one piece with the denture base, not separately printed and waxed. This method permits a direct intraoral esthetic evaluation while maintaining the posterior segments in a simplified form for subsequent adjustments if needed. Subsequently, elastomeric bite registration material (Regisil Rigid, Dentsply Sirona, Charlotte, North Carolina, United States) was used to record the maxillomandibular relationship of the 3D-printed try-in prostheses. It is worth noting that the 3D-printed try-in dentures presented good retention and no reline with PVS was required. After the prosthetic parameters defined with the 3D-printed dentures were deemed adequate, the record was sent back to the manufacturer (AvaDent, Scottsdale, Arizona, United States), and the definitive prostheses were 3D printed with a high-performance photopolymer (Denture 3D+, Next Dent, 3D Systems, Rock Hill, South Carolina, United States) following the contours of the previously validated 3D-printed try-in complete dentures (Figure 9). It is worth mentioning that 0° posterior artificial teeth arranged in a neutrocentric occlusal scheme were used, accounting for the patient's reduced neuromuscular control and severely resorbed mandibular residual ridge. Subsequently, in the fourth appointment, the prostheses were uneventfully delivered after their contours, retention, and fit were verified and approved by the patient and clinician (Figure 10). The definitive CAD–CAM complete dentures in this study were printed entirely as monolithic dentures, with the denture base and teeth fabricated in a single piece using a tooth-colored resin. No separate printing and bonding of the teeth to the base were performed.

## 3. Discussion

In this clinical case series report, the clinical protocols and try-in appliances proposed by AvaDent were implemented with two different restorative materials and manufacturing techniques to treat elderly patients with severely resorbed edentulous ridges. In the first method, try-in white-colored dentures were printed to evaluate esthetics, phonetics, lip support, smile line, retention, and occlusion. In the second method, trial removable prostheses were designed with posterior occlusion rims to simplify registering the centric relation position without posterior interferences, thus minimizing the need for extensive intraoral adjustments given the lack of neuromuscular coordination of the patient. With both techniques, digital s-CAM and a-CAM dentures demonstrated high patient satisfaction in terms of retention and stability, thus improving the quality of life of the patients by combining traditional prosthodontic principles with contemporary digital manufacturing techniques.

In the present clinical reports, protocols involving traditional impression techniques, maxillomandibular relationship records, and articulated definitive casts were implemented. It is worth noting that despite the significant improvements in data acquisition techniques, digital impressions using intraoral scanners (IOSs) present problems when it comes to recording edentulous ridges, particularly in patients with compromised denture base foundations and flabby narrow residual ridges or with a large retruded tongue. These conditions were present in the patients presented and can hinder accurate data acquisition, leading to inaccurate complete denture fabrication [15]. Additionally, the CAD settings related to the digital artificial teeth arrangements can lead to minor inaccuracies after manufacturing, which often require manual adjustments to avoid issues such as undesired anterior occlusal contacts, slides from centric occlusion, and inadequate buccolingual positioning of the maxillary posterior teeth [16, 17]. These adjustments underscore the need for a clinician's and dental laboratory expertise in fine-tuning the digital design to match the patient's specific oral anatomy and newly established occlusion to ensure both functional efficacy and comfort.

A challenge in digital denture fabrication involves the intrinsic mechanical properties of the denture base polymers used and the need for multiple trial fittings to validate the designs intraorally. Unlike conventional dentures where adjustments are made to artificial teeth fixated with wax to the denture bases, digital fabrication may require the creation of several trial dentures using materials like resin or composite, which can complicate the adjustment process [18, 19]. Studies have compared different techniques for fabricating denture bases. Among these techniques, 3D-printed denture bases have shown particularly promising results. Research suggests that 3D printing technology enables superior tissue adaptation compared to conventional and s-CAM bases [18]. This is likely due to the ability of 3D printing to produce more anatomically accurate denture bases that conform more closely to the variations in individual patient anatomy [19]. However, the inferior mechanical properties of most contemporary 3D printing photopolymers demand a meticulous registration of the patients maxillomandibular relationships and centric relation to prevent undesirable complications related to material fracture and accelerated residual ridge resorption triggered by occlusal interferences. The present clinical case series report presents several limitations related to the limited follow-up time and the reduced number of patients treated; furthermore, only one system was used to treat both patients; therefore, other systems may represent favorable alternatives for similar situations and should be considered for different clinical scenarios. Future research focusing on the durability of materials used for 3D printing complete dentures, their long-term stability, and patient-based outcomes across diverse demographics is scarce when compared to other well-established methods such as compression molding and milling.

## 4. Conclusion

The present clinical case series report illustrates two distinct approaches for complete denture fabrication using the AvaDent Milled and Printed System, which combines conventional and digital techniques. The integration of digital technology into conventional methods offers several advantages, including enhanced retention and reduced production time, as evidenced by the satisfactory clinical outcomes presented in this article.

## Data Availability

Data are available from the first author upon request.
